# Genome sequences of *Klebsiella pneumoniae* bacteriophages SF_KL2 and SF_KL25

**DOI:** 10.1128/mra.01211-24

**Published:** 2025-02-06

**Authors:** Sophia Lorenz, Gabriel Abreu, Hugo Oliveira

**Affiliations:** 1 CEB-Centre of Biological Engineering, University of Minho 56059 , Braga, Portugal; 2 LABBELS–Associate Laboratory, Guimarães, Braga, Portugal; University of Maryland School of Medicine, Baltimore, Maryland, USA

**Keywords:** *Klebsiella pneumoniae*, bacteriophages, genome, capsule types, depolymerases

## Abstract

This report describes the genomes of *Klebsiella pneumoniae* phages SF_KL2 and SF_KL25, which infect the encapsulated multidrug-resistant *K. pneumoniae* known to cause nosocomial infections. SF_KL2 and SF_KL25 belong to the genus *Webervirus* (*siphovirus* morphotype) and have genomes of 48,737 and 49,170 bp, respectively. The phages were isolated from wastewater in northern Portugal.

## ANNOUNCEMENT

*Klebsiella pneumoniae* often causes untreatable nosocomial infections in patients with weakened immune systems (1). The difficulty in treatment arises from resistance genes that reduce the efficacy of antibiotics (2). Therefore, through the specific infection of bacterial pathogens, bacteriophage therapy represents an alternative to combat antimicrobial resistance (3).

Phages SF_KL2 and SF_KL25 were isolated from the same enriched wastewater sample collected from a wastewater treatment plant in Braga, Portugal (4). Wild-type strains (H66, MJH599) of *K. pneumoniae* isolated from Hospital of Braga (5) were added to a medium containing an equal volume of sewage water and 2× Tryptic Soy Broth (Sigma-Aldrich) and incubated in a shaker (16 h at 37°C and 120 rpm). After centrifugation (10 min at 4°C and 9,000 × *g*), a volume of the filtered (0.2 µm) supernatant was spotted on bacterial lawns containing the same strains used for enrichment. Phage plaque purification was ensured by amplifying a single phage plaque in new agar plates three times (4). Transmission electron microscopy (TEM) in a Jeol JEM 1400 with uranyl acetate (Sigma-Aldrich) staining evaluated phage morphologies (6). Phage genomic DNA was isolated using the phenol-chloroform (Sigma-Aldrich) method (7), quantified with NanoDrop 1000 Spectrophotometer, and sequenced at Novogene. A VAHTS Universal Plus DNA Library Prep Kit for Illumina (ND617-02) was used to generate a DNA library (350 bp) sequenced on an Illumina Novaseq platform. Briefly, the sheered genomic DNA was ligated with Illumina adapter, amplified by PCR, and size selected. Base calling accuracy was determined by Phred (8). In Geneious Prime version 2019.2.3 (9), raw data were trimmed using BBDuk and sequence reads (4,251,960 and 5,742,711 reads for SF_KL2 and SF_KL25, respectively) were *de novo* assembled using the Geneious *de novo* assembler (medium-low sensitivity setting). PhageTerm version 1.0.12 (10) analyzed phage genome termini. Genomes were annotated with Pharokka version 1.3.2 (11), coupled with Phold version 0.2.0 (12), but also manually annotated using BLASTp (13) (based on the non-redundant protein sequences database) and HHpred (14) (based on the Pfam-A_v36 database with a 10^−5^ < E value < 0). PhageAI version 1.0.2 predicted the phages’ life cycle (https://app.phage.ai/), and PhageDPO version 0.1.0 predicted the existence of depolymerases (bit.ly/phagedpo) (15). Default parameters were mainly used in all bioinformatic tools.

**Fig 1:**
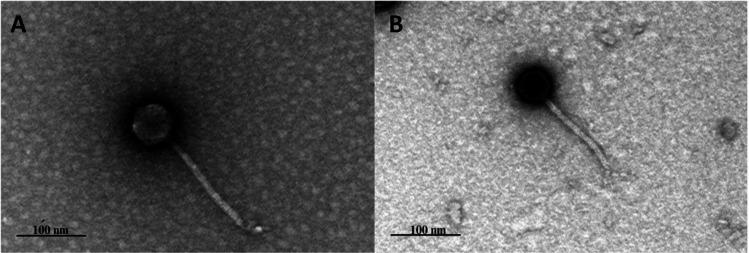
Transmission electron micrograph of *K. pneumoniae* phages. (**A**) SF_KL2 and (**B**) SF_KL25. Phages were stained with 2% uranyl acetate and visualized under a Joel JEM 1400 TEM.

SF_KL2 and SF_KL25 have a *Siphoviridae* morphology, with a non-contractile tail (166 ± 5.1 nm and 161 ± 1.1 nm, respectively) and an icosahedral capsid (55 ± 1.7 nm and 47 ± 2.3 nm, respectively) (Fig. 1). SF_KL2 genome was assembled with 17,450 reads into a 48,737 bp contig (78× coverage), containing 82 coding sequences (CDSs). SF_KL25 genome was assembled with 27,432 reads into a 49,170 bp contig (89× coverage), with 88 CDSs. In both cases, G + C content is 50%, and only 35 proteins have been assigned a function (no tRNA genes). Depolymerases SF_KL2gp65 and SF_KL25gp80 were detected.

According to BLASTn evaluation (13), SF_KL2 and SF_KL25 share high nucleotide identity (>90%) with many other *Klebsiella* phages, though they are most similar to *Weberviruses* PSKP16 (GenBank accession number OW251746) and Sin4 (NC_049847), respectively.

## Data Availability

The genome sequence for phage SF_KL2 is available in the GenBank and SRA, under accession nos. PQ553208 and SRR31221381, respectively. Likewise, the genome sequence for phage SF_KL25 is available in the GenBank and SRA, under accession nos. PQ519586 and SRR31174496, respectively.
